# Optimization of the Protocol for the Isolation and Refolding of the Extracellular Domain of HER2 Expressed in Escherichia coli

**Published:** 2014

**Authors:** V. V. Dolgikh, I. V. Senderskiy, G. V. Tetz, V. V. Tetz

**Affiliations:** Department of Microbiology, Virology and Immunology, First Pavlov State Medical University, of Saint Petersburg, L’va Tolstogo Str., 6-8, 197022, St. Petersburg, Russia

**Keywords:** epidermal growth factor receptor, extracellular domain, bacterial expression, refolding

## Abstract

Receptor 2 of the human epidermal growth factor (HER2/neu, c-erbB2) is a 185 kDa proto-oncogene protein characterized by an overexpression in some oncological diseases, including 30% of mammary glands cancers, as well as tumors in the ovary, stomach and other organs of the human body. Since HER2- tumor status testing is the essential part of a successful cancer treatment, the expression and purification of substantial amounts of the extracellular domain (ECD) of HER2 is an important task. The production of ECD HER2 in* Escherichia coli *has several advantages over the use of eukaryotic expression systems, but the bulk of the recombinant product in bacteria accumulates as insoluble protein inclusion bodies. In this study, we obtained ECD HER2 in *Escherichia coli *as insoluble inclusion bodies and elaborated a simple, efficient, and fast protocol for the solubilization, refolding, and isolation of the protein in soluble form.

## INTRODUCTION

Receptor 2 of the human epidermal growth factor (HER 2/neu, c-erbB2) is a 185 kDa proto-oncogene protein consisting of three main domains: an extracellular, a transmembrane and an intracellular one. The intracellular domain exhibits tyrosine kinase activity [1]. In normal cells, the protein forms heterodimers with some other representatives of the family of human epidermal growth factors and takes part in the regulation of cell proliferation and differentiation [2]. The bulk of oncological diseases, including 30% of mammary gland cancers, as well as tumors in the ovary, stomach, and other organs are accompanied by a hyperexpression of protein HER 2. Herewith, the high level of protein expression is a characteristic feature of cancer recurrence cases with a bad prognosis [2]. A sufficiently effective therapy based on the target drug Herceptin (Trastuzumabum) is used to treat HER 2-positive diseases. New target-based drugs have recently been discovered: Pertuzumab, which inhibits the dimerization of HER 2 with other receptors and immunotoxin Trastuzumab emtansine, which is a conjugate of Herceptin and cytotoxic agent mertansine. Therefore, the immunodiagnostics of the HER 2 status of a tumor is significantly important to a successful treatment. Attention has been paid to revealing HER 2 protein in the serum of patients, together with immunochemical and immunohistological analyses of the material taken at biopsy. It was found that N-terminal part of the molecule presented by the extracellular domain (EC D) of the protein circulates in human blood [3] and that the antibodies specific to this part of the molecule were secreted in the examined patients [4]. A correlation between disease severity and level of EC D HER 2 in serum of patients was evaluated [5]. It was shown later that the dependence is not definitive and that further investigation is warranted for confirmation [6, 7].

The aforesaid data demonstrate the necessity to have at the ready considerable amounts of purified EC D HER 2 that is required for production of diagnostic antibodies and the analysis of the antibodies titer in serum of patients; moreover, it is important for the development of next-generation target drugs [8].

Production of EC D HER 2 in *Escherichia coli *has a number of advantages over the use of eukaryotic expression systems. First of all, the former procedure provides a recombinant product in better yield and lower cost [9]. An additional protocol for production of the relatively inexpensive protein is the expression of EC D HER 2 in yeast *Pichia pastoris*. The process is characterized by protein mannosylation to a high extent, which is not intrinsic in native molecules synthesized in the human body [10]. It should be noted that EC D HER 2 has seven sites for N-glycosylation; therefore, expression of the protein in *Escherichia coli *does not allow one to prepare a protein with the appropriate posttranslational modification. However, the protein produced by the bacteria is of indubitable interest for the elaboration of test systems and screening of suitable antibodies. Accumulation of the recombinant product in the form of insoluble inclusion bodies is also an essential disadvantage in the expression of EC D HER 2 in cells of* Escherichia coli*.

Thus, heterology expression sequence encoding EC D HER 2 (together with the signal peptide) on pGEX-6P-1 and pQE30 vectors allowed one to prepare recombinant proteins bound to the N-terminal groups of glutathione S-transferase (GST-EC D HER 2) and a sequence consisting of six histidine residues, respectively [9]. In both cases, almost all the expression product accumulated in bacterial cells in the form of insoluble inclusion bodies. The authors tried different schemes for refolding with a varied pH, temperature, incubation time, concentrations of urea, EDTA (ethylenediaminetetraacetic acid), L-arginine, oxidized, and reduced glytathione. All these modifications had only insignificant impact on the efficiency of GST-EC D HER 2 refolding. Thus, the efficiency was 63–92% of the best achieved value for some of the protocols applied. The authors emphasized that only two of the seven analyzed factors, namely, pH and incubation time, influenced the efficiency of protein refolding. This fact allowed us to propose that recombinant EC D HER 2 in a form of protein inclusion bodies accumulated in bacteria may be converted to soluble species via the simple procedures of dilution, concentration, and dialysis.

In our previous study, we repeated the expression of EC D HER 2 in *Escherichia coli *using pRSET vector (Life Technologies) under the control of a highly active promoter, bacteriophage T7 [11]. In the construct used, the N-terminal signal peptide responsible for the secretion of HER 2 and cut in a eukaryotic cell in the course of its translocation into the endoplasmic reticulum lumen, was removed. In this case, the vector construction implies the addition of the N-terminal peptide with a polyhistidine sequence to the recombinant protein. In this study, the recombinant protein obtained in a form of insoluble protein inclusion bodies was used to demonstrate the possibility of its solubilization and refolding by the most simple and fast protocol, without the use of complex reagents. We also demonstrated the crucial role of SH-reagents for effective dissolution of the protein inclusion bodies formed by EC D HER 2.

## EXPERIMENTAL

The procedures for cloning the sequence encoding human EC D HER 2 in vector pRSET , as well as the effective production of the recombinant protein in strain C41 *E. coli*, isolation, washing off and storage of protein inclusion bodies were described earlier [11].

The approaches used to solubilize and restore the native conformation of the recombinant protein are listed in the Results and Discussion section.

The protein was purified by metal-chelate affinity chromatography on a column with a HIS-Select® resin (Sigma-Aldrich) equilibrated by a solution containing 50 mM Tris-HCl (pH 8), 0.3 M NaCl, and 10 mM imidazole. For this purpose, a solution of the protein containing the same components was passed through the column. The bound components were washed with the equilibration buffer solution and eluted in the presence of 0.3 M imidazole. The resulting fractions were combined, concentrated using Centricon centrifugal concentrators (Millipore) that allowed passage of molecules of up to 30 kDa through them, and stored at –20 °C in the presence of a 50% glycerol solution. The protein concentration in the samples was determined by the Bradford method [12].

Gel electrophoresis of the proteins in a 12% polyacrylamide gel in the presence of an anionic detergent, sodium dodecyl sulfate (SDS-PAGE), was performed in a Mini-PROTE AN® chamber (Bio-Rad). For immunoblotting, the proteins after SDS-PAGE were transferred on a nitro-cellulose membrane using a Mini-Trans- Blot® insert according to the manufacturer’s instructions. The membranes were blocked for 1 h in the presence of TT BS (50 mM Tris-HCl (pH 7.4), 150 mM NaCl and 0.05% Tween 20 solution) and a 1% BSA solution; they were then incubated overnight at 4°C with anti-polyHis-antibodies (Sigma-Aldrich) conjugated to horseradish peroxidase (the antibodies were diluted 3,000-fold by the same solution). After washing off in TT BS and then in TBS (TT BS without Tween 20), the membrane was incubated in a freshly prepared solution for developing the peroxidase reaction containing TBS, 15% methanol, 0.05% 4-chloro-1-naphthol (Sigma- Aldrich), and 0.02% H_2_O_2_.

## RESULTS AND DISCUSSION

At the first stage of our study, the recombinant protein EC D HER 2 produced in bacteria was isolated as insoluble protein inclusion bodies according to the earlier described procedure [11]. In order to solubilize the protein inclusion bodies, the scheme used for GST-EC D HER 2 [9] was repeated, strictly following the reported protocol. The protein inclusion bodies were re-suspended in a solution containing 10 mM Tris-HCl, 0.1 M NaH2PO4, 8 M urea, and 5 mM dithiothreitol (pH 8) and were incubated for 30 min at room temperature. After centrifugation of the solubilized material at 14,000 g for 10 min pellet was re-suspended in the same solution until the volume of supernatant was reached. The content of the recombinant protein in the pellet and the supernatant was compared by SDS-PAGE. The experiments showed that approximately half of the recombinant protein remained insoluble after the described scheme was used. Moreover, the achieved result was observed only when a freshly prepared solution was used. When stored for ~ 2 months at 4 °C, the efficiency of the solution for solubilization of the protein significantly decreased. It was proposed that the main reason for the decrease in the solubilizing property of the solution might be due to the oxidation of dithiotreitol, which is a relatively unstable reagent containing reduced SHgroups.

**Fig. 1:**
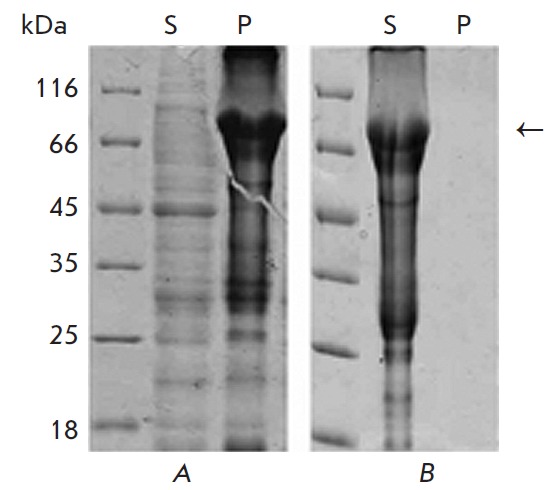
Analysis of the precipitates (P) and supernatants (S) after extraction of the recombinant ECD HER2 from the protein inclusion bodies. Solutions applied: A – 50 mM Tris-HCl (pH 8), 8 M urea; B – 50 mM Tris-HCl (pH 8), 8 M urea, 1% 2-mercaptoethanol. The proteins were separated by SDS-PAGE in a 12% gel and stained with a Coomassie R-250 dye. The bands corresponding to the investigated protein are shown with an arrow

To confirm the important role of SH-reagents in protein solubilization, we analyzed the solubility of the recombinant product in the presence of 8 M urea without addition of SH-containing reagents, as well as in the presence of 8 M urea with 1% 2-mercaptoethanol added. With this aim in mind, the protein inclusion bodies were solubilized in a solution containing 50 mM Tris- HCl (pH 8), together with the aforementioned components, and the solution was incubated for 30 min at room temperature. Identically to the previous experiment, the solubilized material was separated by centrifugation and the resulting pellets were re-suspended in the same solutions until they reached the volume of the supernatant in order to compare the content of the recombinant protein in the samples. The experiment showed that the EC D HER 2 accumulated in bacteria was practically insoluble in the solution containing 8 M urea without 2-mercaptoethanol (*Fig. 1A*). When 2-mercaptoethanol was added to the solubilizing solution, total dissolution of the recombinant protein was registered (*Fig. 1B*). This result confirmed the crucial role of SH-containing compounds in the solubilization of EC D HER 2 expressed in bacteria and proved that the increased content of dithiotreitol or 2-mercaptoethenol in the solution for the solubilization of protein inclusion bodies can increase the yield of the soluble form of EC D HER 2. A quantitative determination of the protein content in the supernatant demonstrated that the protocol used allowed us to obtain about 70 mg of the protein extracted from the protein inclusion bodies, which were isolated from 1 L of bacteria.

The material extracted in the presence of 8 M urea and 1% solution of 2-mercaptoethanol was used to as sess the possibility of using the simplest scheme for refolding when producing recombinant EC D HER 2 in the soluble form. For this purpose, the supernatant from the previous experiment containing 18 mg/ml of the solubilized protein was diluted 250-fold under vigorous stirring. The solution for dilution was the same as that applied to equilibrate a HIS-Select® Ni-containing resin (Sigma-Aldrich) during metal chelate affinity chromatography (50 mM Tris-HCl (pH 8.0), 0.3 M NaCl and 10 mM imidazole). The protein diluted in 50 ml of solution was immediately passed through a column with 400 μl of the resin; the column was thoroughly washed with the equilibration buffer, and the recombinant EC D HER 2 was eluted in the presence of a 0.3 M imidazole solution. The protein content measured in the starting extract solution and the resulting fractions showed that 15 % of the protein loaded into the column was bound to the resin and eluted (0.54 of 3.6 mg).

**Fig. 2:**
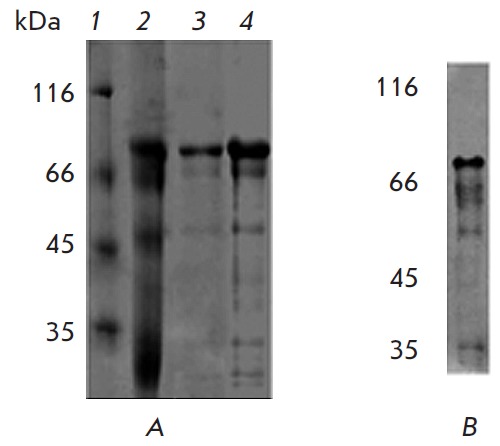
Analysis of the soluble form of ECD HER2 by SDS-PAGE and immunoblotting technique. A – The protein extracted in the presence of 8 M urea and 1% 2-mercaptoethanol (track 2) was diluted 250-fold with the equilibration buffer resin HIS-Select® and purified on a column using metal chelate affinity chromatography (lanes 3 and 4, different amounts of the protein were applied). The proteins were separated by SDS-PAGE in a 12% gel and stained with a Coomassie R-250 dye. Track 1 is plotted on the molecular weight markers. B – Immunoblotting analysis of the protein extracted from the protein inclusion bodies demonstrated the presence of minor bands of a smaller size recognized by anti-polyhistidine antibodies, which attested to an insignificant hydrolysis of the recombinant protein

The analysis of the fractions obtained by SDS-PAGE proved that the protocol applied allowed us to prepare the soluble recombinant protein in a sufficiently pure form (*Fig. 2A*, lanes 3 and 4). The resulting fractions contained almost no ballast proteins with a molecular weight higher than that of EC D HER 2. Some minor components with a lower molecular weight might result from an insignificant extent of the hydrolysis of recombinant protein during its isolation, since some of them react with anti-polyhistidine antibodies (*Fig. 2B*). Since the protein purified by metal chelate affinity chromatography most often contains some impurities, optimization of the chromatography conditions and the use of additional purification methods may be helpful for obtaining products completely free of impurities.

## Abbreviations

ECD HER2: extracellular domain of receptor 2 of the human epidermal growth factor
SDSPAGE: protein electrophoresis in polyacrylamide gel in the presence of sodium dodecyl sulfate as an anionic detergent.

## References

[1] Coussens L., Yang-Feng T.L., Liao Y.C., Chen E., Gray A., McGrath J., Seeburg P.H., Libermann T.A., Schlessinger J., Francke U. (1985). Tyrosine kinase receptor with extensive homology to EGF receptor shares chromosomal location with neu oncogene.. Science..

[2] Marmor M.D., Skaria K.B., Yarden Y. (2004). Signal transduction and oncogenesis by ErbB/HER receptors.. Int. J. Radiat. Oncol. Biol. Phys..

[3] Tan M., Yu D. (2007). Molecular mechanisms of erbB2-mediated breast cancer chemoresistance.. Adv. Exp. Med. Biol..

[4] Santin A.D., Bellone S., Roman J.J., McKenney J.K., Pecorelli S. (2008). Trastuzumab treatment in patients with advanced or recurrent endometrial carcinoma overexpressing HER 2/neu.. Int. J. Gynaecol. Obstet..

[5] Menard S., Balsari A., Casalini P., Tagliabue E., Campiglio M., Bufalino R., Cascinelli N. S. (2002). HER -2-positive breast carcinomas as a particular subset with peculiar clinical behavior. Clin. Cancer Res..

[6] Sandri M.T., Johansson H., Colleoni M., Zorzino L., Passerini R., Orlando L., Viale G. (2004). Serum levels of HER 2 EC D can determine the response rate to low dose oral cyclophosphamide and methotrexate in patients with advanced stage breast carcinoma.. Anticancer Res..

[7] Disis M.L., SchiVman K., Guthrie K., Salazar L.G., Knutson K.L., Goodell V., dela Rosa C., Cheever M.A. (1996). Effect of dose on immune response in patients vaccinated with an her-2/neu intracellular domain protein-based vaccine.. J. Clin. Oncol..

[8] Pichon M.F., Hacene K., Guepratte S., Neumann R. (2004). Serum HER -2 extracellular domain (EC D) before the first metastasis in 128 breast cancer. Clin. Lab..

[9] Leary A.F., Hanna W.M., van de Vijver M.J., Penault-Llorca F., Rüschoff J., Osamura R.Y., Bilous M., Dowsett M. (2009). Serum HER -2 extracellular domain (EC D) before the first metastasis in 128 breast cancer patients.. Clin. Lab..

[10] Lennon S., Barton C., Banken L., Gianni L., Marty M., Baselga J., Leyland-Jones B. (2009). Utility of serum HER 2 extracellular domain assessment in clinical decision making: pooled analysis of four trials of trastuzumab in metastatic breast cancer.. J Clin Oncol..

[11] Belimezi M.M., Papanastassiou D., Merkouri E., Baxevanis C.N., Mamalaki A. (2006). Growth inhibition of breast cancer cell lines overexpressing Her2/neu by a novel internalized fully human Fab antibody fragment.. Cancer Immunol. Immunother..

[12] Liu X., He Z., Zhou M., Yang F., Lv H., Yu Y., Chen Z. (2007). Purification and characterization of recombinant extracellular domain of human HER 2 from Escherichia coli.. Protein Expr. Purif..

[13] Dimitriadis A., Gontinou C., Baxevanis C.N., Mamalaki A. (2009). The mannosylated extracellular domain of Her2/neu produced in P. pastoris induces protective antitumor immunity.. BMC Cancer..

[14] Waugh D.S. (2005). Making the most of affinity tags.. Trends Biotechnol..

[15] Nallamsetty S., Waugh D. (2006). Solubility-enhancing proteins MBP and NusA play a passive role in the folding of their fusion partners.. Protein Expr. Purif..

[16] Nallamsetty S., Waugh D.S. (2007). A generic protocol for the expression and purification of recombinant proteins in Escherichia coli using a combinatorial His6-maltose binding protein fusion tag.. Nature Protocols.

[17] Dolgikh V.V., Senderskiy I.V., Tetz G.V., Tetz V.V. (2013). Extracellular domain of HER 2 heterologous expression in bacteria. Accepted for publication by Uchenye zapiski Sankt-Peterburguskogo Gosudarstvennogo Meditsinskogo Universiteta im. acad. I.P. Pavlova (Proceedings of St. Petersburg State Pavlov Medical University) in September 2013 (in Russian)..

[18] Vogelstein B., Gillespie D. (1979). Proc. Natl. Acad. Sci. USA..

[19] Miroux B., Walker J.E. (1996). J. Mol. Biol..

[20] Laemmli U.K. (1970). Nature.

[21] Cleland W.W. (1964). Biochemistry..

